# Scaffold protein GhMORG1 enhances the resistance of cotton to *Fusarium oxysporum* by facilitating the MKK6‐MPK4 cascade

**DOI:** 10.1111/pbi.13307

**Published:** 2019-12-20

**Authors:** Chen Wang, Hongbin Guo, Xiaowen He, Shuxin Zhang, Jiayu Wang, Lijun Wang, Dezheng Guo, Xingqi Guo

**Affiliations:** ^1^ State Key Laboratory of Crop Biology College of Life Sciences Shandong Agricultural University Taian China; ^2^ Statistics Department University of Auckland Auckland New Zealand; ^3^ State Key Laboratory of Crop Biology Shandong Agricultural University Taian China

**Keywords:** MAPK scaffold protein, phosphoproteomics, disease resistance, *Gossypium hirsutum*

## Abstract

In eukaryotes, MAPK scaffold proteins are crucial for regulating the function of MAPK cascades. However, only a few MAPK scaffold proteins have been reported in plants, and the molecular mechanism through which scaffold proteins regulate the function of the MAPK cascade remains poorly understood. Here, we identified GhMORG1, a GhMKK6‐GhMPK4 cascade scaffold protein that positively regulates the resistance of cotton to *Fusarium oxysporum*. GhMORG1 interacted with GhMKK6 and GhMPK4, and the overexpression of *GhMORG1* in cotton protoplasts dramatically increased the activity of the GhMKK6‐GhMPK4 cascade. Quantitative phosphoproteomics was used to clarify the mechanism of GhMORG1 in regulating disease resistance, and thirty‐two proteins were considered as the putative substrates of the GhMORG1‐dependent GhMKK6‐GhMPK4 cascade. These putative substrates were involved in multiple disease resistance processes, such as cellular amino acid metabolic processes, calcium ion binding and RNA binding. The kinase assays verified that most of the putative substrates were phosphorylated by the GhMKK6‐GhMPK4 cascade. For functional analysis, nine putative substrates were silenced in cotton, respectively. The resistance of cotton to *F. oxysporum* was decreased in the substrate‐silenced cottons. These results suggest that GhMORG1 regulates several different disease resistance processes by facilitating the phosphorylation of GhMKK6‐GhMPK4 cascade substrates. Taken together, these findings reveal a new plant MAPK scaffold protein and provide insights into the mechanism of plant resistance to pathogens.

## Introduction

During their growth and development, plants are inevitably subjected to various environmental stresses. Plants perceive and transduce environmental and developmental cues intracellularly via a set of interconnected intracellular signalling networks (Guo *et al.*, 2016). Evolutionarily conserved mitogen‐activated protein kinase (MAPK) cascades are among the most thoroughly studied signalling cascades (Fiil *et al.*, 2009). Plant MAPK cascades are composed of three sequentially tiered protein kinases. MAPKs are phosphorylated and activated by upstream MAPK kinases (MKKs; alternatively MAPKKs and MEKs). The activation of MKKs is regulated by the topmost component of the cascade, MKK kinases (MAPKKKs; alternatively MEKKs), via phosphorylation (MAPK Group *et al.*, 2002). In the last two decades, numerous studies have demonstrated the important role of MAPK cascades in plant immunity. The activation of MAPK cascades is one of the earliest events during the plant immune response and controls both the biosynthesis of phytoalexins and the expression of defence‐related genes (Bi and Zhou, 2017; Meng and Zhang, 2013). MEKK1‐MKK4/MKK5‐MAPK3/MAPK6 was one of the earliest MAPK cascades identified and functions downstream of the flagellin receptor FLS2 (Asai *et al.*, 2002). The activation of this MAPK cascade can provide resistance to both bacterial and fungal pathogens in *Arabidopsis* by activating defence‐related gene expression, phytoalexin biosynthesis and stomatal immunity (Li *et al.*, 2012; Su *et al.*, 2017; Xu *et al.*, 2016). Another well‐studied disease resistance‐related MAPK cascade is composed of MEKK1, MKK1/MKK2 and MAPK4, which regulates the accumulation of ROS and salicylic acid (SA) (Gao *et al.*, 2008; Pitzschke *et al.*, 2009).

MKK6 is one of the most widely studied MAPKK proteins. In *Arabidopsis*, MKK6 is required for regulating cytokinesis as part of the ANPs‐MKK6‐MPK4 cascade (Kosetsu *et al.*, 2010). However, recent studies have reported that MKK6 can be phosphorylated by MAPKKK5 and is involved in MAPKKK5‐mediated resistance to both bacterial and fungal pathogens (Yan *et al.*, 2018). In cotton, the MKK6‐mediated MAPK cascade also plays an important role in plant immunity (Wang *et al.*, 2017). These studies suggest that the function of MKK6 is very complex, and a key question regarding this MAPK cascade is the mechanism by which the different functions of this pathway can be regulated via a common set of components.

In mammals, MAPK scaffold proteins play important roles in regulating the crosstalk of MAPK cascades (Meister *et al.*, 2013). Scaffold proteins contribute to the efficiency and specificity of signal transmission by binding several MAPK components (Yoshioka, 2004). The yeast protein Ste5 was the first reported MAPK scaffold protein linking the MAPK cascade to G protein signalling in the mating pathway (Witzel *et al.*, 2012). In plants, few proteins have been shown to be scaffold proteins of MAPK. In *Arabidopsis*, breaking of asymmetry in the stomatal lineage (BASL) and 14‐3‐3ω act as MAPK scaffold proteins that regulate the different functions of the MAPK3/MAPK6‐mediated MAPK cascade (Guo *et al.*, 2016; Zhang *et al.*, 2015). BASL regulates MAPK3/MAPK6‐mediated stomatal development (Zhang *et al.*, 2015), whereas 14‐3‐3ω is involved in chloroplast retrograde signalling by coupling Ca^2+^ signalling (released by chloroplasts) and the MAPK cascade (Guo *et al.*, 2016).

The WD40 domain repeat protein is a large family in plants. The WD40 domain is a short structural motif of approximately 40 amino acids that terminates with tryptophan–aspartic acid (W‐D) (Neer *et al.*, 1994). These domains usually form the β‐propeller, which is a molecular platform that facilitates interactions with several ligands from distinct signalling pathways at the same time (Islas‐Flores *et al.*, 2015). Receptor for activated C kinase 1 (RACK1), a typical WD40 domain repeat protein, was the first reported MAPK scaffold protein associated with the plant immune response (Su *et al.*, 2015). In response to *Pseudomonas aeruginosa*, RACK1 interacts with Gβ, MEKK1, MKK4/MKK5 and MAPK3/MAPK6 to form a complex in *Arabidopsis* (Cheng *et al.*, 2015).

Previous studies have shown that MAPK scaffold proteins mainly affect MAPK cascades upon the perception of different external stimuli. However, the molecular mechanism by which the MAPK scaffold protein regulates the function of the MAPK cascade remains poorly understood. Different signals may direct cell‐specific MAPKs to interact with different substrates, thus leading to different physiological events (Zhang *et al.*, 2016). MAPK scaffold proteins may regulate the different functions of the MAPK cascade by phosphorylating different substrates. The gaps of knowledge in plant MAPK cascades are caused by a lack of information regarding MAPK substrates. Phosphorylation is a very important posttranslational modification (PTM), and protein phosphorylation can affect the expression of downstream genes (Li *et al.*, 2015), protein turnover (Liu and Zhang, 2004), and other biological processes by altering protein stability, enzyme activity (Liu and Zhang, 2004), and subcellular localization (Roux *et al.*, 2015). Although multiple MAPK substrates have been identified by the phosphoproteome in the last decade (Sörensson *et al.*, 2012), these substrates do not fully explain the complicated function of MAPK cascades.

Cotton is an important economic crop worldwide. Nevertheless, various cotton diseases severely threaten cotton production (Xie *et al.*, 2015). Fusarium wilt, which is caused by the fungus *Fusarium oxysporum* f. sp. *vasinfectum*, is one of the most severe cotton diseases (Gaspar *et al.*, 2014). Therefore, it is of great practical significance to improve the resistance of cotton to *F. oxysporum*. In our previous studies, we reported that the GhMKK6‐mediated MAPK cascade plays a positive role in cotton immunity. Silencing *GhMKK6* significantly decreased the resistance of cotton to *F. oxysporum* (Wang *et al.*, 2017). In the present study, to analyse the regulatory mechanism of the GhMKK6‐mediated MAPK cascade, we used GhMKK6 as a bait protein to screen interacting proteins in a cotton cDNA library via yeast two‐hybrid assays. A WD40 domain repeat protein, GhMORG1, was identified and determined to be a scaffold protein of the GhMKK6‐GhMPK4 cascade. Using quantitative phosphoproteomics, we found 32 putative substrates of the GhMORG1‐dependent GhMKK6‐GhMPK4 cascade involved in the resistance of cotton to *F. oxysporum*. The functional analysis of the putative substrates revealed that the GhMORG1‐dependent GhMKK6‐GhMPK4 cascade increased the resistance of cotton to *F. oxysporum* by regulating gene transcription and translation, Ca^2+^‐dependent pathways, and H^+^‐ATPase activity. Our study reports a new plant MAPK scaffold protein, GhMORG1, and reveals the mechanism of the GhMORG1‐dependent GhMKK6‐GhMPK4 cascade in regulating the resistance of cotton to *F. oxysporum*.

## Results

### GhMORG1 interacts with GhMKK6

A previous study reported that the GhMKK6‐mediated MAPK cascade increased cotton resistance to *F. oxysporum* by regulating SA‐ or jasmonic acid (JA)‐mediated defence pathways (Wang *et al.*, 2017). To elucidate the molecular mechanism by which GhMKK6 regulates plant disease resistance, GhMKK6 was used as the bait to perform a yeast two‐hybrid screen of the cotton cDNA library. CotAD_19088 and CotAD_21502 (gene IDs in the Cotton Genome Project database) were identified as candidate proteins that interact with GhMKK6. Database queries and bioinformatic analyses revealed that CotAD_19088 (designated GhMPK4) is a MAPK protein that is highly homologous to AtMPK4 (At4G01370). In model plants, MPK4 is the downstream component of MKK6 in *Arabidopsis* (Lian *et al.*, 2018).

**Figure 1:**
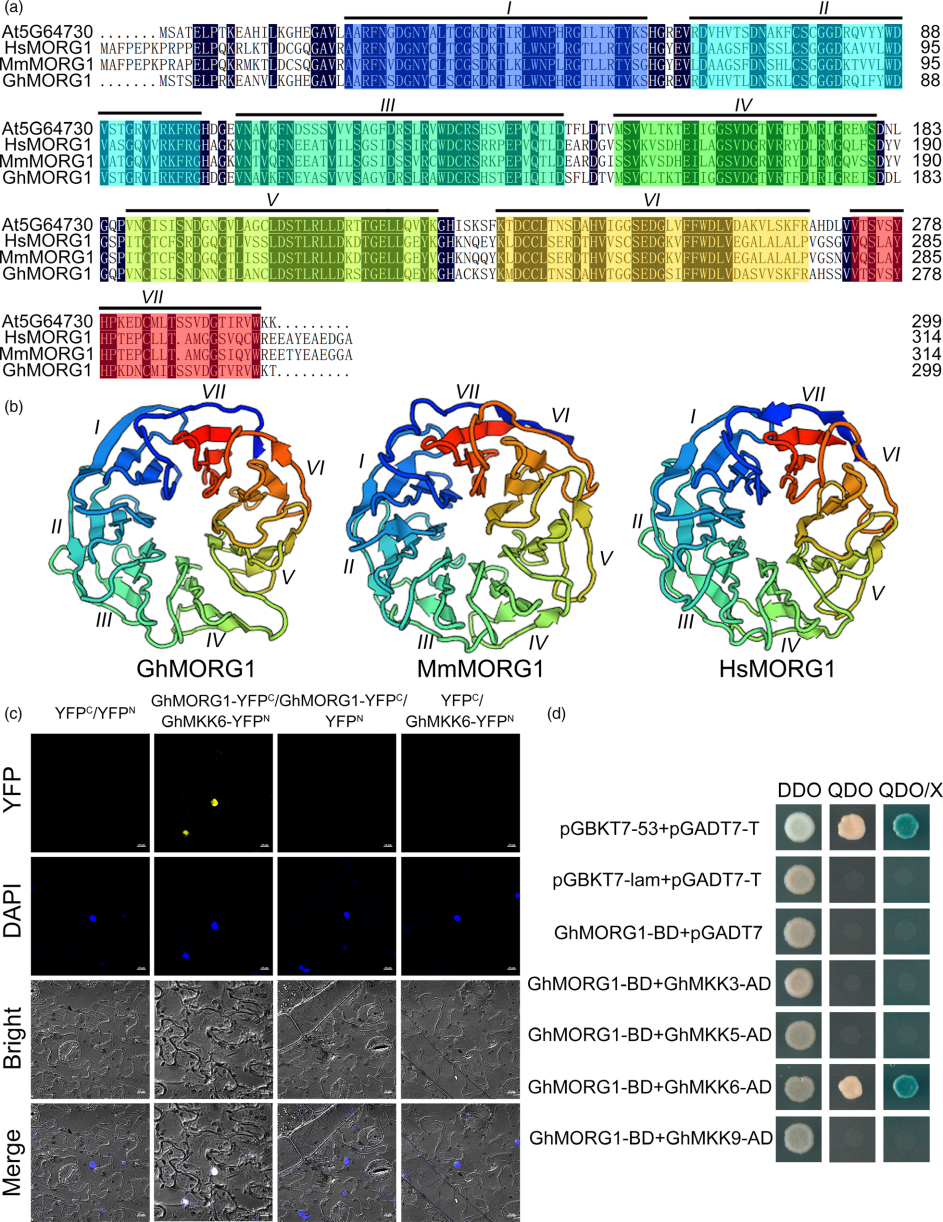
Characterization of GhMORG1 and the interaction between GhMORG1 and GhMKK6. (a) Multiple amino acid sequence alignment of GhMORG1, At5G64730, HsMORG1 (NP_115708) and MmMORG1 (NP_080675). The Roman numerals (I‐VII) at the top indicate seven tandem WD40 domains. (b) SWISS‐MODEL‐predicted tertiary structure of GhMORG1. (c) The interaction between GhMORG1 and GhMKK6 was confirmed by BiFC experiments. Yellow fluorescence was observed with an LSM 880 META confocal microscope (Carl Zeiss). (d) GhMORG1 specifically interacts with GhMKK6 according to the yeast two‐hybrid results. The indicated BD and AD fusion constructs were cotransformed into yeast and grown on DDO, QDO and QDO/X SD media.

*CotAD_21502* consists of 900 bp and encodes a 299‐amino acid protein. A sequence analysis showed that CotAD_21502 shares high homology not only with At5G64730 from *Arabidopsis* but also with mitogen‐activated protein kinase organizer 1 (MORG1) (NP_080675) from *Mus musculus* and MORG1 (NP_115708) from *Homo sapiens* (Figure 1a). Therefore, *CotAD_21502* was designated *GhMORG1*. Similar to MmMORG1 and HsMORG1, the deduced amino acid sequence of GhMORG1 was found to contain seven tandem WD40 domains that form β‐propeller structures (Figure 1b) (Meister *et al.*, 2013; Xu and Min, 2011).

The bimolecular fluorescence complementation (BiFC) system was used to verify the interaction between GhMKK6 and GhMORG1 *in vivo*. GhMKK6‐YFP^N^ and GhMORG1‐YFP^C^ were cotransformed into *Nicotiana benthamiana* leaves via agroinfiltration, and fluorescent signals were detected (Figure 1c). To determine whether GhMORG1 specifically interacted with GhMKK6, a yeast two‐hybrid system was used to detect the interactions between GhMORG1 and cotton MKKs (Figure S1). The results showed that only the positive control clone and the clone cotransformed with GhMKK6 and GhMORG1 grew well on synthetically defined (SD) medium without Leu and Trp (DDO), SD medium without Ade, His, Leu, and Trp (QDO), and QDO/X (QDO with X‐a‐gal) medium (Figure 1d). These results indicated that GhMORG1 specifically interacted with GhMKK6.

### GhMORG1 positively regulates cotton resistance to *F. oxysporum*

**Figure 2:**
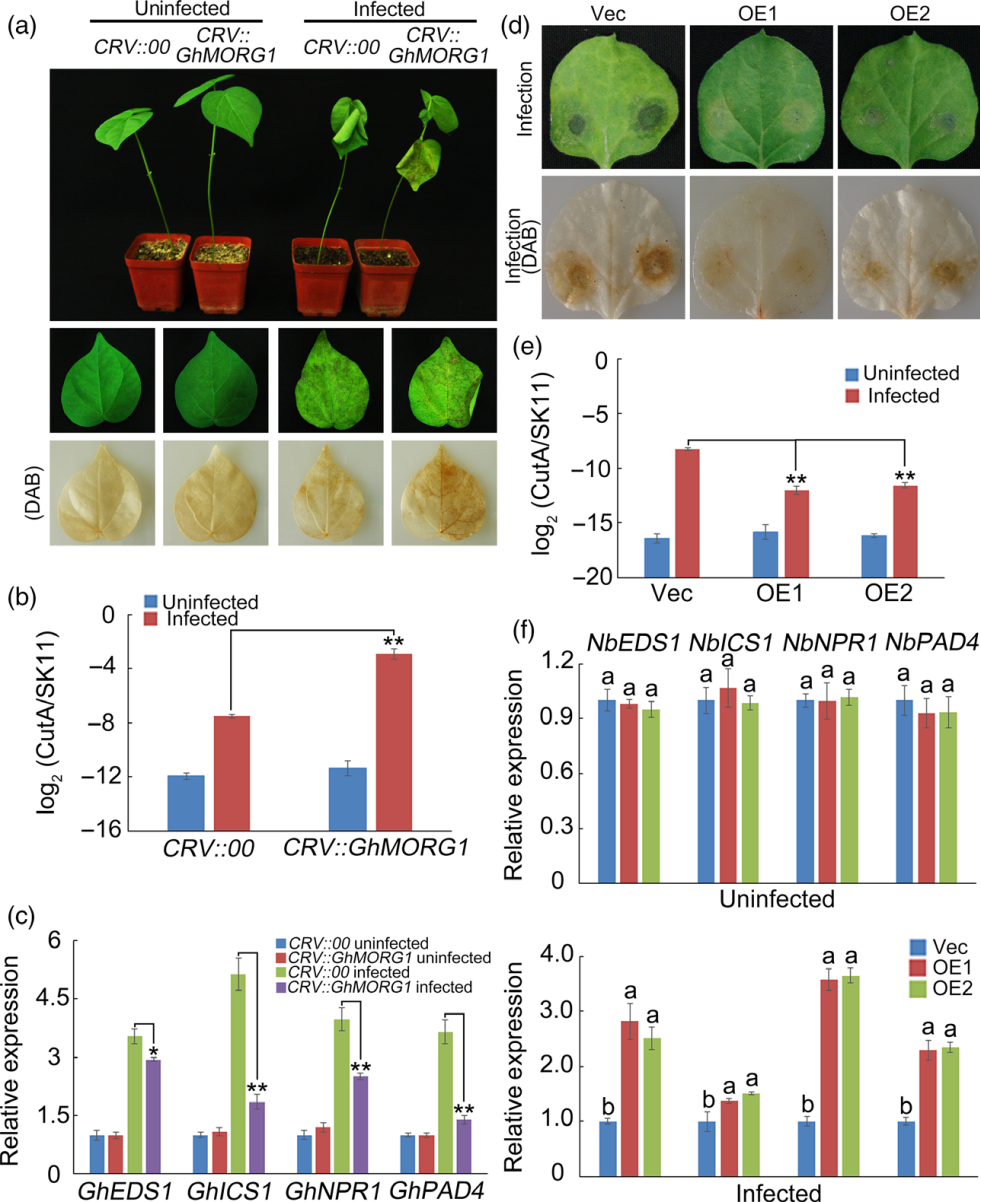
*GhMORG1* positively regulated the resistance of cotton to *F. oxysporum*. (a) Representative phenotypes of *GhMORG1*‐silenced cotton plants after five days of *F. oxysporum* infection. (b) Pathogen disease index in *GhMORG1*‐silenced cotton after five days of *F. oxysporum* infection. (c) Expression levels of SA‐mediated defence pathway genes in CRV::00 and CRV::GhMORG1 cotton. (d) Representative phenotypes of *GhMORG1*‐overexpressing tobacco after *F. oxysporum* infection. (e) Pathogen disease index in *GhMORG1*‐overexpressing tobacco after *F. oxysporum* infection. (f) Expression levels of SA‐mediated defence pathway genes in *GhMORG1*‐overexpressing tobacco. The error bars in (b, c and e) indicate the mean ± SE of three independent experiments (*n* = 15). Asterisks above the lines indicate significant differences (*P < 0.05, **P < 0.01) based on Tukey’s HSD test. Data in (f) are means ± SE of three independent experiments (*n* = 15). Different letters indicate significant differences (*P* < 0.01) based on Tukey’s HSD test.

To investigate the function of *GhMORG1* in plant resistance to pathogens, *Agrobacterium*‐mediated virus‐induced gene silencing (VIGS) was used to silence *GhMORG1* in cotton. Three weeks after *Agrobacterium* infiltration, the expression level of *GhMORG1* was significantly reduced (Figure S2a). The cotton leaf crumple virus‐based vector (CRV)::00 (empty vector control) and CRV::GhMORG1 plants were root‐wounded and then infected with an *F. oxysporum* spore suspension (10^6^ conidia/mL). Five days after inoculation, the CRV::GhMORG1 leaves showed more severe signs of chlorosis than the CRV::00 leaves (Figure 2a and Figure S3a). The pathogen disease index of the CRV::GhMORG1 leaves was much higher than that of the CRV::00 leaves (Figure 2b and Figure S3b). We also detected the expression levels of genes involved in SA‐mediated defence pathways (Figure 2c and Figure S3c). As shown in Figures 2c and Figure S3c, the expression levels of SA‐related genes were much lower in the CRV::GhMORG1 leaves than in the CRV::00 leaves after *F. oxysporum* infection.

Then, *GhMORG1* was introduced into *N. benthamiana* using the *A. tumefaciens*‐mediated leaf disc method, and GhMORG1‐overexpressing transgenic *N. benthamiana* (OE) was obtained (Figure S2b,c). A six‐week‐old empty vector control line (Vec) and two independent OE lines (OE1 and OE2) (the T_2_ generations) were used to analyse the function of *GhMORG1* in plant disease resistance. After *F. oxysporum* inoculation for five days, the degree of morbidity of leaves from OE lines was less than that in the Vec line (Figure 2d). The pathogen disease index of the OE leaves was lower than that of the Vec line leaves, and the genes involved in SA‐mediated defence pathways showed higher expression levels in the OE lines than in the Vec line after *F. oxysporum* infection (Figure 2e,f). These results showed that *GhMORG1* positively regulates the resistance of cotton to *F. oxysporum*.

### As a scaffold protein, GhMORG1 enhances the activity of the GhMKK6‐GhMPK4 cascade

**Figure 3:**
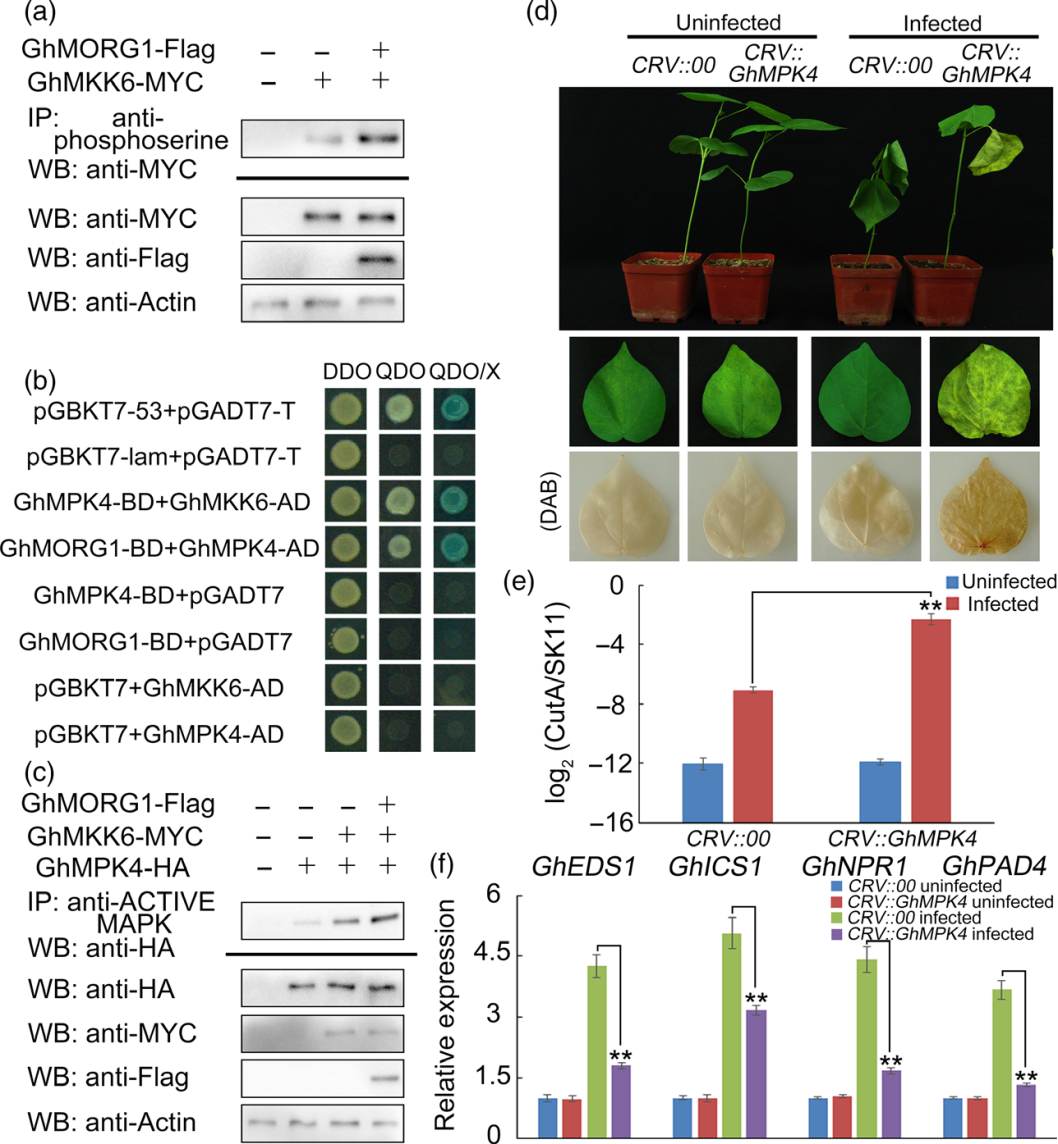
GhMPK4 interacts with GhMORG1 and GhMKK6 and plays important roles in the resistance of cotton to *F. oxysporum*. (a) The phosphorylation level of GhMKK6 in cotton protoplasts coexpressing GhMORG1 and GhMKK6. (b) GhMPK4 interacts with GhMKK6 and GhMORG1 according to the yeast two‐hybrid results. (c) The phosphorylation level of GhMPK4 in cotton protoplasts coexpressing GhMORG1, GhMKK6 and GhMPK4. (d) Representative phenotypes of *GhMPK4*‐silenced cotton after five days of *F. oxysporum* infection. (e) Pathogen disease index in *GhMPK4*‐silenced cotton after five days of *F. oxysporum* infection. (f) Expression levels of SA‐mediated defence pathway genes in CRV::00 and CRV::GhMPK4 cotton. The error bars indicate the mean ± SE of three independent experiments (*n* = 15). Asterisks above the lines indicate significant differences (*P < 0.05, **P < 0.01) based on Tukey’s HSD test.

To evaluate whether GhMORG1 affected the activity of GhMKK6, GhMORG1 and GhMKK6 were coexpressed in cotton protoplasts. The anti‐phosphoserine antibody was used to immunoprecipitate all proteins for which the serine residue was phosphorylated. As shown in Figure 3a, the accumulation of phosphorylated GhMKK6 was dramatically increased in the protoplasts coexpressing GhMORG1 and GhMKK6.

The yeast two‐hybrid screen showed that GhMPK4 was a potential interacting protein of GhMKK6. As shown in Figure 3b, the yeast two‐hybrid assay indicated that the growth of the clone cotransformed with GhMKK6 and GhMPK4 or cotransformed with GhMPK4 and GhMORG1 and the growth of the positive control clone were similar. The BiFC system was used to verify the interaction between GhMPK4 and GhMKK6 or GhMORG1 *in vivo*. Fluorescent signals were detected in the *N. benthamiana* leaves that were cotransformed with GhMKK6‐YFP^N^ and GhMPK4‐YFP^C^ or cotransformed with GhMPK4‐YFP^N^ and GhMORG1‐YFP^C^ (Figure S4). These results demonstrate that GhMPK4 interacts with GhMKK6 and GhMORG1. To determine whether GhMORG1 affected the activity of the GhMKK6‐GhMPK4 cascade, GhMORG1, GhMKK6 and GhMPK4 were coexpressed in cotton protoplasts. As shown in Figure 3c, the accumulation of phosphorylated GhMPK4 was dramatically higher in the protoplasts coexpressing GhMKK6 and GhMPK4 than in the protoplasts cotransformed with empty vector and 35S::GhMPK4‐HA after all of the phosphorylated MAPK was immunoprecipitated by the anti‐pTEpY phospho‐p44/42 MAPK antibody. Furthermore, the phosphorylation level of GhMPK4 was higher in the protoplasts coexpressing GhMORG1, GhMKK6 and GhMPK4 than in the protoplasts coexpressing GhMKK6 and GhMPK4 (Figure 3c). This result indicated that GhMORG1 enhanced the activity of the GhMKK6‐GhMPK4 cascade.

To test the functions of *GhMPK4* in the cotton immune response, we used VIGS to silence *GhMPK4* in cotton. CRV::00 (empty vector control) and CRV::GhMPK4 plants were root‐wounded and then infected with a *F. oxysporum* spore suspension (10^6^ conidia ml^–1^). After five days of *F. oxysporum* infection, the CRV::GhMPK4 leaves showed more severe signs of chlorosis than the CRV::00 leaves (Figure 3d and Figure S5a). The pathogen disease index of the CRV::GhMPK4 leaves was much higher than that of the CRV::00 leaves, and the SA‐mediated genes exhibited the same expression profiles between the *GhMORG1*‐silenced cotton and *GhMPK4*‐silenced cotton (Figures 3e,f and Figure S5b,c). These results indicated that GhMORG1 regulated the resistance of cotton to *F. oxysporum* by mediating the GhMKK6‐GhMPK4 cascade.

### Proteome‐wide analysis of phosphorylated proteins in GhMORG1‐silenced cotton in response to *F. oxysporum* infection

**Figure 4:**
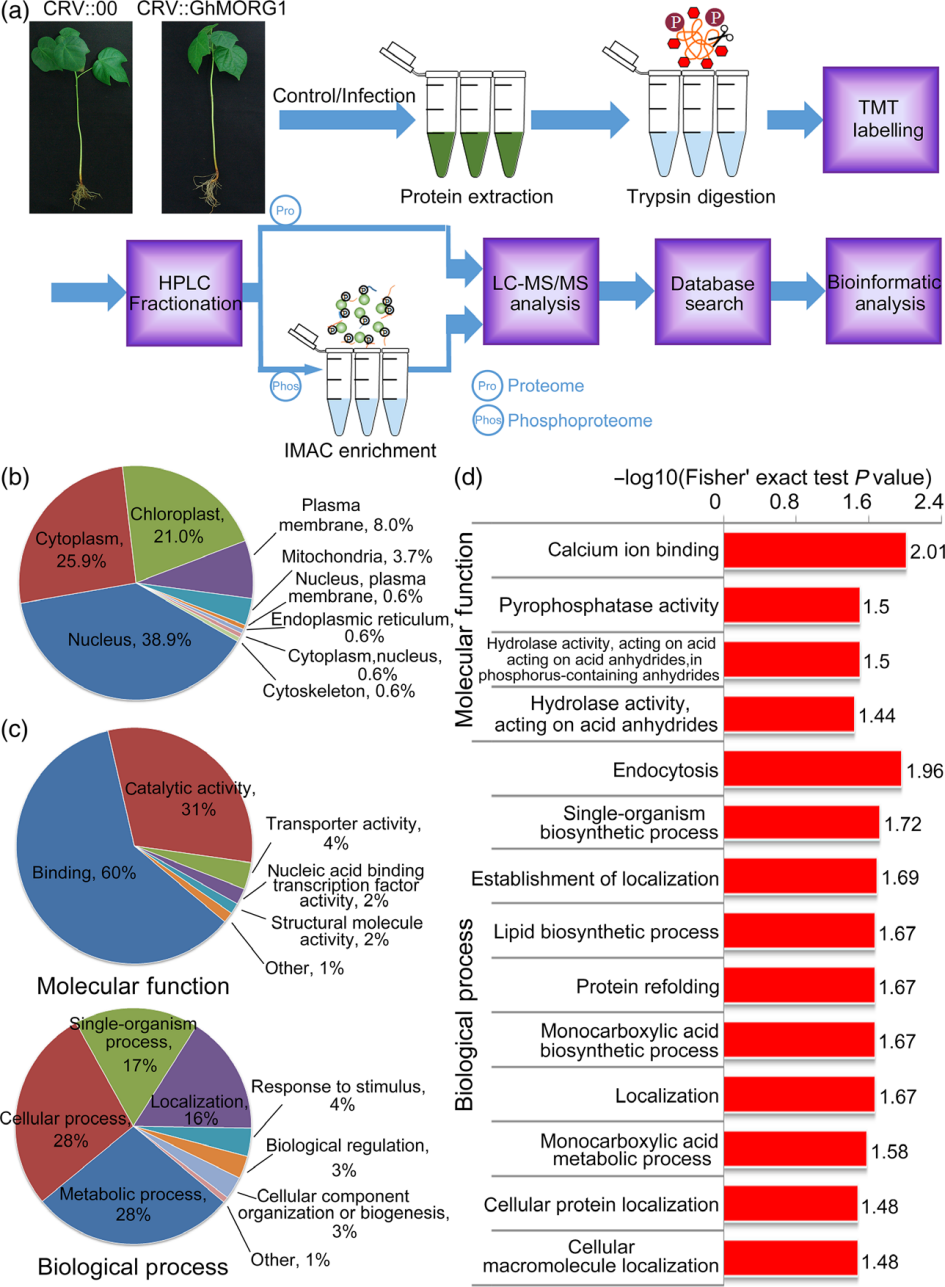
GO‐based enrichment analyses of increased‐ and decreased‐regulated phosphorylated proteins in response to *F. oxysporum* infection in cotton. (a) Systematic workflow of the quantitative profiling of the global phosphoproteome in CRV::00 and CRV::GhMORG1 cotton infected or uninfected with *F. oxysporum*. (b) Subcellular localization of increased‐ and decreased‐regulated phosphorylated proteins by GO analysis in CRV::00 cotton after *F. oxysporum* infection. (c, d) GO‐based enrichment analysis of the increased‐ and decreased‐regulated phosphorylated proteins associated with biological process and molecular function in CRV::00 cotton after *F. oxysporum* infection.

To clarify the molecular mechanism underlying the ability of GhMORG1 to regulate the cotton immune response mediated by the GhMKK6‐GhMPK4 cascade, the phosphopeptides isolated from CRV::00 and CRV::GhMORG1 cotton with or without *F. oxysporum* infection were compared. A proteomic analysis was then performed to quantify protein abundance in each cotton genotype and treatment (Figure 4a). Three independent biological replicates were analysed per cotton genotype and treatment (Figure S6). The proteomic analysis identified 9053 proteins from cotton, and 7376 proteins were quantified (Table S1). In addition, 5065 phosphorylation sites in 2920 proteins were identified, among which 3421 sites in 2134 proteins were quantified (Table S2). To determine the motifs of the identified phosphorylation sites, the Motif‐X program was used to compare the position‐specific frequencies of the amino acid residues. Among the 5065 identified phosphorylation sites in this study, 39 conserved motifs among 3977 sites were identified, accounting for approximately 78.5% of the identified phosphorylation sites (Table S3). The six most abundant motifs were designated......SP.....,...R..S......,...R..SP.....,.L.R..S......,......SPR.... and......TP..... (. indicates any amino acid) (Figure S7).

To analyse the effect of *F. oxysporum* infection on cottons, we compared the phosphorylation levels of proteins from CRV::00 cottons that were infected or were uninfected with *F. oxysporum*. The results showed that a total of 184 phosphorylation sites among 150 proteins presented at least a 1.5‐fold increase and that 14 phosphorylation sites among 14 proteins presented at least a 1.5‐fold decrease (the data from proteomics were used for normalization) (*P* < 0.05) (Table S4). To elucidate the functions of the proteins exhibiting an increase or decrease in phosphorylation, Gene Ontology (GO) enrichment‐based analyses were performed. On the basis of the subcellular classification analysis, the largest proportion was assigned to the nucleus, followed by the cytoplasm and chloroplast (Figure 4b). In the molecular function analysis, binding and catalytic activity were the most enriched terms for the proteins exhibiting an increase and a decrease in phosphorylation, which mainly affected calcium ion binding (GO:0005509) and pyrophosphatase activity (GO:0016462) (Figure 4c,d, Table S5). In the biological process category, proteins exhibiting an increase and a decrease in phosphorylation were enriched mainly in the metabolic process, cellular process, single‐organism process and localization and mainly affected endocytosis (GO:0006897), single‐organism biosynthetic process (GO:0044711) and establishment of localization (GO:0051234) (Figure 4c,d, Table S5).

**Figure 5:**
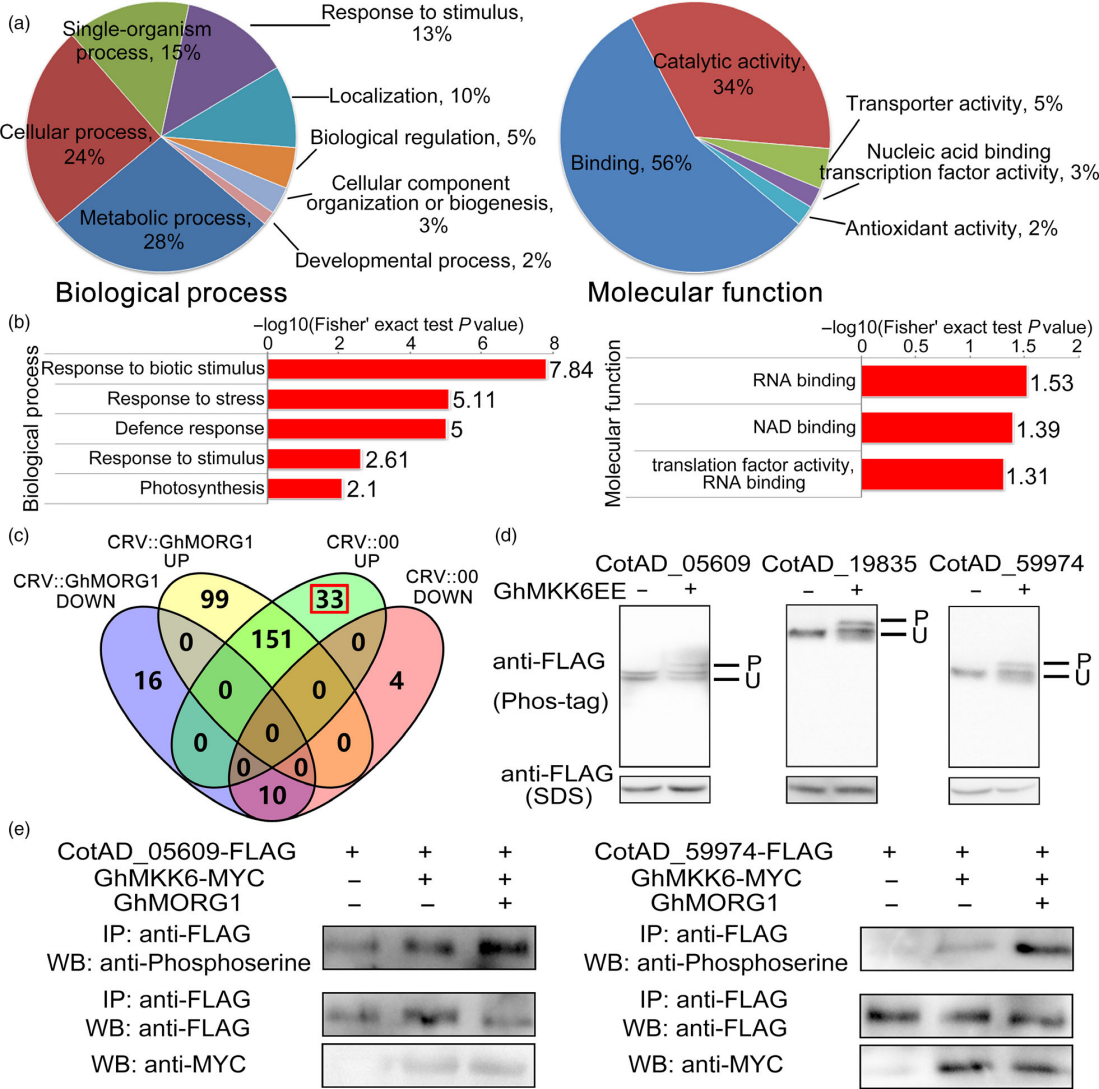
Screening of putative substrates of the GhMORG1‐dependent GhMKK6‐GhMPK4 cascade. (a, b) GO‐based enrichment analysis of the increased‐ and decreased‐regulated phosphorylated proteins from CRV::00 and CRV::GhMORG1 cotton infected with *F. oxysporum*. (c) Venn diagram illustrating the overlap of increased‐ or decreased‐regulated phosphopeptides identified in CRV::00 and CRV::GhMORG1 cotton after *F. oxysporum* infection. CRV::GhMORG1 up, increased phosphorylated proteins from CRV::GhMORG1 cotton infected with *F. oxysporum*. CRV::GhMORG1 down, decreased phosphorylated proteins from CRV::GhMORG1 cotton infected with *F. oxysporum*. CRV::00 up, increased phosphorylated proteins from CRV::00 cotton infected with *F. oxysporum*. CRV::00 down, decreased phosphorylated proteins from CRV::00 cotton infected with *F. oxysporum*. (d) Phosphorylation level of candidate putative substrates in cotton protoplasts. At the top of each panel, the characters indicate the Gene ID in the Cotton Genome Project database. P and U indicate the phosphorylated and unphosphorylated forms, respectively. (e) Phosphorylation level of candidate substrates in cotton protoplasts coexpressing GhMKK6, GhMORG1 and substrates.

To determine the function of *GhMORG1* in cotton disease resistance, we compared the phosphorylated proteins between CRV::00 and CRV::GhMORG1 cotton after *F. oxysporum* infection; 56 proteins showed at least a 1.5‐fold increase, and 3 proteins showed at least a 1.5‐fold decrease (CRV::GhMORG1 infection vs CRV::00 infection) (*P* < 0.05) (Table S6). Based on the GO enrichment analysis, in the molecular function category, proteins exhibiting an increase and a decrease in phosphorylation were most enriched in binding and catalytic activity and mainly affected the terms RNA binding (GO:0003723), NAD binding (GO:0051287) and translation factor activity (GO:0008135) (Figure 5a,b, Table S7). In the biological process category, the proteins exhibiting an increase and a decrease in phosphorylation were most enriched in metabolic process and cellular process and mainly affected the terms response to biotic stimulus (GO:0009607), response to stress (GO:0006950) and defence response (GO:0006952) (Figure 5a,b, Table S7). These results suggested that GhMORG1 regulated multiple disease resistance‐related processes in cotton.

### Identification of GhMORG1‐mediated GhMKK6‐GhMPK4 cascade substrates

The phosphopeptides that increased in response to *F. oxysporum* infection in CRV::00 cotton but that were not detected in CRV::GhMORG1 cotton were considered putative substrates of the *F. oxysporum*‐induced GhMORG1‐dependent GhMKK6‐GhMPK4 cascade. As shown in Figure 5c and Table S8, we revealed 33 phosphorylation sites in 32 proteins that may be the substrates of GhMORG1‐dependent GhMKK6‐GhMPK4 cascade in response to *F. oxysporum* infection.

To confirm that the putative substrates could be phosphorylated by GhMKK6, we randomly selected 8 of the 32 proteins based on the ratio of the increased phosphorylation (CRV::00 infection vs CRV::00) (Table S8), and the candidate proteins were, respectively, coexpressed with activated GhMKK6 (GhMKK6EE) in cotton protoplasts. The cotton protoplast coexpressed candidate proteins and empty vector were used as the controls. Phos‐tag technology was used to detect the phosphorylation level of the selected substrates. As shown in Figure 5d and Figure S8a, all of the selected proteins could be phosphorylated by activated GhMKK6. Then, the substrates, GhMKK6 and GhMORG1 were coexpressed in cotton protoplasts. The results in Figure 5e and Figure S8b showed that GhMORG1 increased the candidate substrate phosphorylation which was mediated by GhMKK6. Furthermore, the yeast two‐hybrid assay indicated that the growth of the clone cotransformed with GhMPK4 and candidate proteins (CotAD_29344, CotAD_42472, CotAD_59974) and the growth of the positive control clone were similar (Figure S9a). We also detected three putative substrates that could be phosphorylated by activated GhMPK4 (GhMPK4GA). As shown in Figure S9b, the phosphorylation level of the selected proteins was altered in the protoplasts that expressed GhMPK4GA. These results implied that most of the 32 proteins were substrates of the GhMORG1‐dependent GhMKK6‐GhMPK4 cascade.

**Figure 6:**
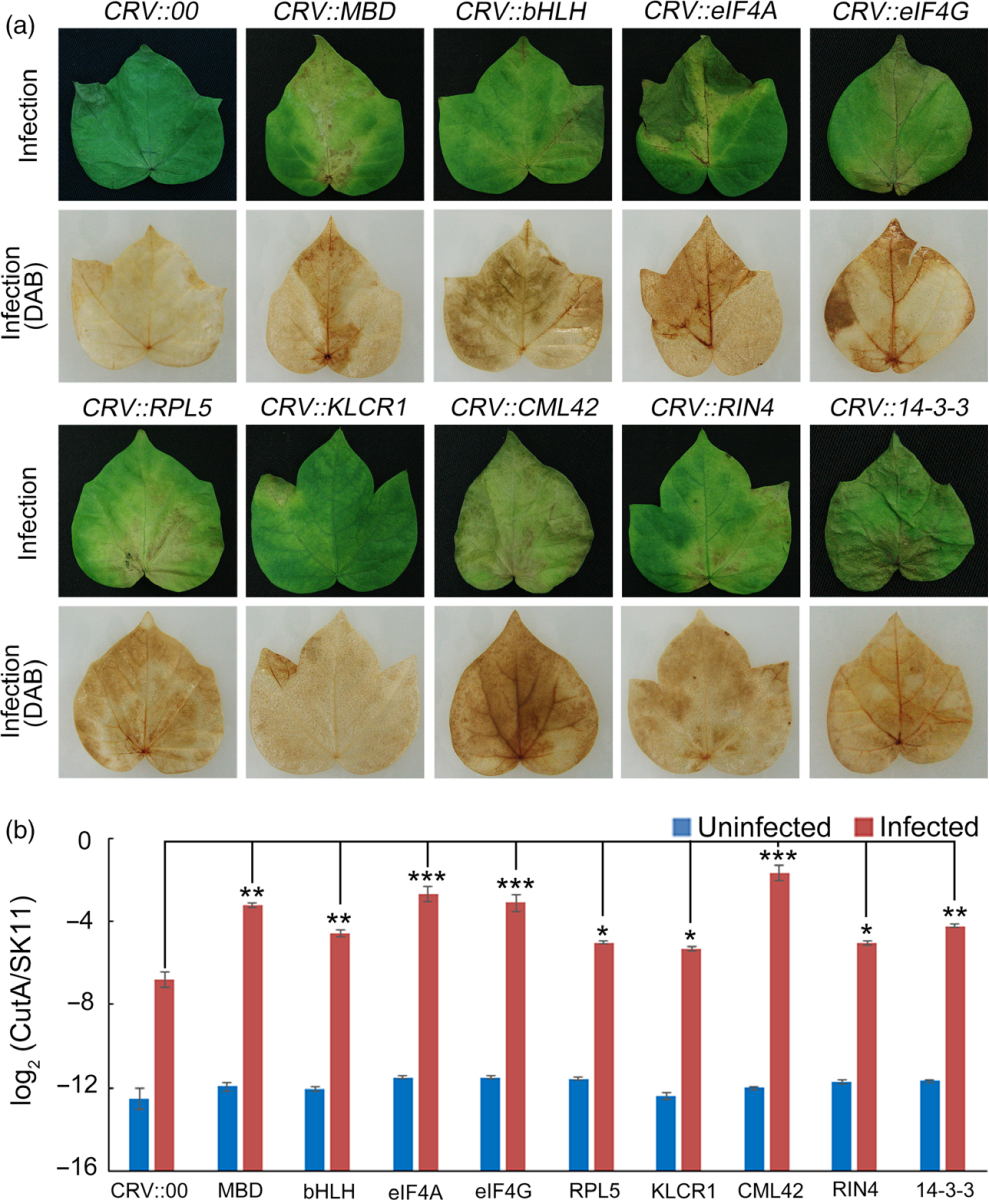
Functional analysis of GhMORG1‐dependent GhMKK6‐GhMPK4 cascade substrates. (a) Representative phenotypes of different substrate‐silenced cotton after five days of *F. oxysporum* infection. (b) Pathogen disease index in different substrate‐silenced cotton after five days of *F. oxysporum* infection. Data are means ± SE of three independent experiments (*n* = 9). Asterisks (*, ** or ***) above lines indicate significant differences (*P < 0.05; **P < 0.01; ***P < 0.001) based on Tukey’s HSD test.

To determine whether the putative substrates were involved in regulating the resistance of cotton to *F. oxysporum*, 9 of 32 putative substrate genes were silenced in cotton using VIGS, respectively. After five days of *F. oxysporum* infection, the leaves of CRV::MBD (CotAD_33461), CRV::bHLH (CotAD_59974), CRV::eIF4A (CotAD_53993), CRV::eIF4G (CotAD_56516), CRV::RPL5 (CotAD_29344), CRV::KLCR1 (CotAD_19835), CRV::CML42 (CotAD_35634), CRV::RIN4 (CotAD_42472) and CRV::14‐3‐3 (CotAD_64613) showed more severe signs of chlorosis than those of the control plants (CRV::00) (Figure 6a). The pathogen disease index of the substrate‐silenced cotton leaves was markedly higher than that of the CRV::00 leaves (Figure 6b). These results indicated that most of the putative substrates played important roles in regulating the resistance of cotton to *F. oxysporum*.

## Discussion

In eukaryotes, the role of scaffold proteins in regulating the MAPK cascade has been well established. In mammals, MORG1 is a partner of the extracellular signal‐regulated kinase (ERK) pathway scaffold protein MP1 and interacts specifically with several components of the ERK pathway (such as RAF‐1, MEK and ERK) (Meister *et al.*, 2013; Vomastek *et al.*, 2004). MORG1 can facilitate lysophosphatidic acid‐induced ERK activation but does not respond to epidermal growth factors (Vomastek *et al.*, 2004). In plants, MAPK scaffold proteins have received widespread attention in recent years. Moreover, 14‐3‐3ω was recently identified as a Ca^2+^‐dependent MAPK scaffold protein in *Arabidopsis*, linking chloroplast‐modulated Ca^2+^ signalling to the MAPK cascade during retrograde responses (Guo *et al.*, 2016). The down‐regulation of *14‐3‐3ω* expression reduced the activation of the MAPK3/MAPK6‐mediated MAPK cascade (Guo *et al.*, 2016). RACK1 is a member of the WD40 domain repeat protein family and can also form β‐propeller structures (Miller *et al.*, 2016). Cheng *et al. *(2015) reported that RACK1, as a MAPK scaffold protein, links the MAPK cascade to an upstream G protein to mediate *P. aeruginosa*‐triggered immunity (Cheng *et al.*, 2015). Silencing RACK1 via artificial microRNA markedly reduced the levels of activated MAPK3 and MAPK6 (Cheng *et al.*, 2015). In this study, we identified a WD40 domain repeat protein, GhMORG1, in cotton (Figure 1). GhMORG1 played a positive role in the resistance of cotton to *F. oxysporum* (Figures 2 and Figure S3). Furthermore, GhMORG1 interacts specifically with the GhMKK6‐GhMPK4 cascade. MAPKKKs were the topmost component of the MAPK cascade, and MAPK cascade scaffold proteins often interacted with all the three levels of MAPK, MAPKK and MAPKKK. Unfortunately, some reported MAPKKKs, such as MAPKKK5, could not interact with GhMORG1. This result indicated that there might be a new MAPKKK gene regulating the function of GhMKK6‐GhMPK4 cascade in cotton. More importantly, overexpressing GhMORG1 in cotton protoplasts dramatically increased the phosphorylation level of GhMKK6‐GhMPK4 cascade (Figure 3). Therefore, we speculated that GhMORG1 was a new plant MAPK scaffold protein that regulated GhMKK6‐GhMPK4 cascade‐mediated cotton immunity.

Phosphorylation is a widely used mechanism of PTM (Wang *et al.*, 2013). The MAPK scaffold protein regulated the function of the MAPK cascade by affecting the phosphorylation levels of MAPKs and substrates. Our global phosphoproteomic analysis revealed that GhMORG1, as the scaffold protein of the GhMKK6‐GhMPK4 cascade, involved in multiple disease resistance‐related processes by regulating the phosphorylation levels of substrates of the GhMKK6‐GhMPK4 cascade (Figures 4 and 5). Although we have not demonstrated the putative substrates were specifically phosphorylated by GhMKK6‐GhMPK4 cascade, the kinase assay revealed that most of the putative substrates could be phosphorylated by GhMKK6 or GhMPK4.

Previous studies have reported that MAPK cascades can regulate transcription and translation by phosphorylating their substrates (Pitzschke, 2015). In plants, the family of bHLH transcription factors is widespread and plays essential roles in plant disease resistance by regulating gene transcription (Goossens *et al.*, 2017). Ribosomal protein RPL5 is a ribosomal component and is important for the recruitment of 5S rRNA, the assembly of the large ribosomal subunit and the maintenance of the reading frame during translation. Decreased RPL5 levels in plants putatively lead to impaired translation (Korepanov *et al.*, 2007; Moradi *et al.*, 2008). eIFs control translation initiation, which is the rate‐limiting step of translation (Holcik and Sonenberg, 2005). eIF4A interacts with eIF4G, and stress signalling suppresses translation initiation by interfering with the interaction between eIF4E and eIF4G (Jackson *et al.*, 2010). In this study, based on database queries and bioinformatic analysis, we identified bHLH122 (CotAD_59974), RPL5 (CotAD_29344), eIF4A (CotAD_53993) and eIF4G (CotAD_56516, CotAD_57266) as putative substrates of the GhMORG1‐dependent GhMKK6‐GhMPK4 cascade. Silencing bHLH122, RPL5, eIF4A and eIF4G decreased the resistance of cotton to *F. oxysporum* (Figure 6). These observations suggested that the GhMORG1‐dependent GhMKK6‐GhMPK4 cascade activated plant immunity by regulating gene transcription and translation.

Ca^2+^ is a key secondary messenger in plants and plays a pivotal role in the regulation of numerous developmental processes and responses to a myriad of abiotic cues and biotic challenges (DeFalco *et al.*, 2009; Dodd *et al.*, 2010; Reddy *et al.*, 2011). Although the MAPK cascade and Ca^2+^‐mediated signalling pathway are involved in regulating plant disease resistance, the relationship between the two pathways remains unclear. Calmodulin‐like protein (CML) 42 (CotAD_35634) and kinesin light chain‐related protein‐1 (KLCR1) (CotAD_19835), which are crucial regulatory factors in Ca^2+^ signalling (Bürstenbinder *et al.*, 2013; Vadassery *et al.*, 2012), were identified as putative substrates of the GhMORG1‐dependent GhMKK6‐GhMPK4 cascade and participated in the resistance of cotton to *F. oxysporum* (Figure 6). Furthermore, CotAD_30923, which encodes a cysteine proteinase inhibitor, was identified as a substrate and involved in regulating Ca^2+^‐dependent pathways (Guo *et al.*, 2013). These results suggested that GhMORG1 linked the MAPK cascade with calcium‐mediated disease resistance signalling pathways by regulating the phosphorylation of GhMKK6‐GhMPK4 cascade.

Based on the GO enrichment analysis, the increased‐ and decreased‐regulated phosphorylated proteins from CRV::GhMORG1 cotton mainly affected the term ATPase activity (GO:0016887). We also found that cotton RPM1‐interacting protein 4 (RIN4) (CotAD_42472) and 14‐3‐3 protein (CotAD_64613) are putative substrates of the GhMORG1‐dependent GhMKK6‐GhMPK4 cascade, which is involved in regulating the resistance of cotton to *F. oxysporum* (Figure 6). It was recently reported that RIN4 interacts with GCN4 (an AAA^+^‐ATPase) and 14‐3‐3 proteins and regulates plasma membrane H^+^‐ATPase activity in *Arabidopsis* (Kaundal *et al.*, 2017). The overexpression of GCN4 resulted in the degradation of both RIN4 and 14‐3‐3 proteins via the proteasome pathway and reduced the stomatal response to fusicoccin (a fungal toxin produced by the fungus *Fusicoccum amygdali*) (Kaundal *et al.*, 2017). These results suggested that the GhMORG1‐mediated MAPK cascade affected the cotton response to *F. oxysporum* by regulating plasma membrane H^+^‐ATPase activity.

**Figure 7:**
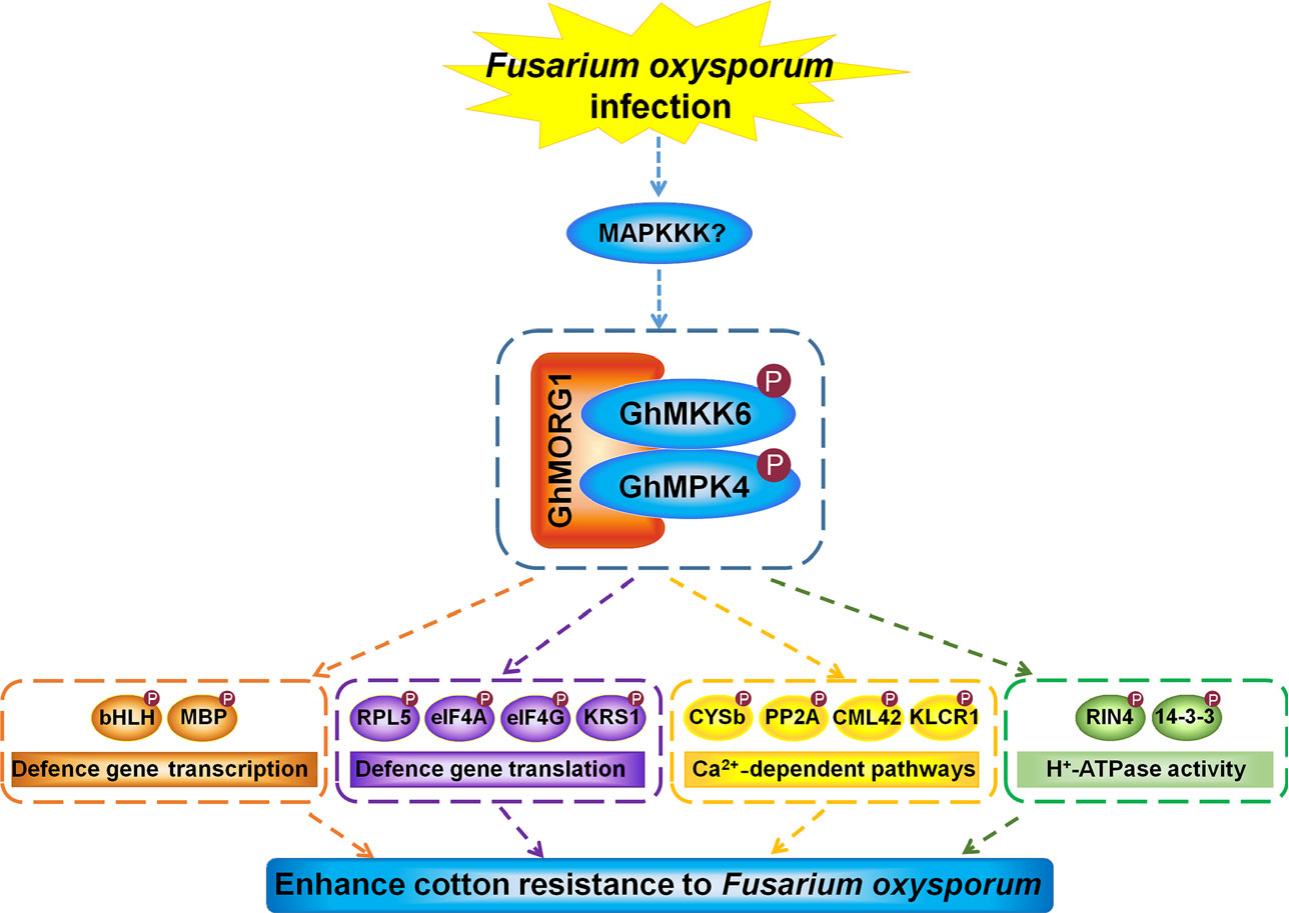
Diagram depicting the function of substrates of the GhMORG1‐dependent GhMKK6‐GhMPK4 cascade in this study. After *F. oxysporum* infection, the MAPK scaffold protein GhMORG1‐mediated GhMKK6‐GhMPK4 cascade was activated. Based on our results, we speculated that this pathway increased the resistance of cotton to *F. oxysporum* by regulating gene transcription and translation, Ca^2+^‐dependent pathways, and H^+^‐ATPase activity.

In conclusion, the MKK6‐mediated MAPK cascade plays important roles in plant immunity. In this study, GhMORG1, a GhMKK6‐GhMPK4 cascade scaffold protein, was identified in cotton via a yeast two‐hybrid screen. Silencing *GhMORG1* significantly reduced the resistance of cotton to *F. oxysporum*. A quantitative phosphoproteomic analysis identified 32 putative substrates of the GhMORG1‐dependent GhMKK6‐GhMPK4 cascade. The overexpression of *GhMORG1* increased the phosphorylation of some putative substrates, mediated by the GhMKK6‐GhMPK4 cascade. Silencing most of the putative substrates dramatically decreased the resistance of cotton to *F. oxysporum*. The functional analysis of the putative substrates revealed that the GhMORG1‐dependent GhMKK6‐GhMPK4 cascade increased the resistance of cotton to *F. oxysporum* by regulating gene transcription and translation, Ca^2+^‐dependent pathways, and H^+^‐ATPase activity (Figure 7). Although we have demonstrated the role of some substrate proteins in the resistance of cotton to *F. oxysporum*, the regulatory mechanism of the GhMORG1‐dependent GhMKK6‐GhMPK4 cascade on substrate proteins still requires further study. The provided data set identified a new MAPK scaffold protein and may serve as an important resource for the functional analysis of the MAPK cascade, which may facilitate the elucidation of the regulatory mechanism of MAPK cascades in response to pathogens.

## Experimental procedures

### Plant materials, fungal strains and growth conditions

Cotton seeds (*Gossypium hirsutum* L. cv. lumian 22) were germinated on wet gauze at 28 ± 1 °C. The germinated seedlings were grown under greenhouse conditions (28 ± 1 °C temperature, 16‐h light/8‐h dark cycle and 60%–75% relative humidity). *N. benthamiana* seeds were surface‐sterilized and germinated on Murashige–Skoog (MS) medium under greenhouse conditions (25 ± 1 °C temperatures, 16‐h light/8‐h dark cycle). At the three‐leaf stage, the plants were transplanted into soil. *F. oxysporum* (strain AYF‐1, from the Institute of Cotton Research of CAAS) was grown on potato dextrose agar (PDA) at 25 °C. The pathogenicity of this strain was previously described (Pei *et al.*, 2019). For *F. oxysporum* treatment in cottons, three‐and‐a‐half‐week‐old cotton plants were root‐wounded and then infected with an *F. oxysporum* spore suspension (10^6^ conidia/mL). The *F. oxysporum* treatment in tobacco consisted of injecting the leaves with 100 μL of conidial *F. oxysporum* suspensions (10^6^ conidia/mL) using a syringe. The leaves from the treated cotton plants or the treated tobacco leaves were collected and frozen in liquid nitrogen for further analyses. Each treatment was repeated at least three times.

### Gene cloning, bioinformatic analysis and genetic transformation

The open reading frame (ORF) of *GhMORG1* was isolated from a cotton cDNA library via PCR. The primers used are listed in Table S9. Full‐length *GhMORG1* ORF was introduced into tobaccos using the *Agrobacterium tumefaciens*‐mediated leaf disc method (Lu *et al.*, 2013). The homologous GhMORG1 proteins were aligned using DNAMAN 5.2.2 software (Lynnon Biosoft). The tertiary structures were predicted using SWISS‐MODEL (http://swiss-model.expasy.org/).

### Yeast two‐hybrid screening and bimolecular fluorescence complementation (BiFC)

To identify the interacting proteins of GhMKK6, GhMKK6 was used as the bait in a yeast two‐hybrid screen of the cotton cDNA library. The experiment was performed using the Matchmaker Gold Yeast Two‐Hybrid System (Clontech, Japan) according to the manufacturer’s recommended protocol. After removing the duplicate, untranslated, failed‐sequencing fragments, we obtained candidate proteins that interacted with GhMKK6.

With respect to the yeast two‐hybrid system, the ORF of *GhMORG1* was cloned and inserted into the pGBKT7 vector. The ORFs of cotton MKKs (the A group MKK gene *GhMKK6*, B group MKK gene *GhMKK3* [GenBank accession number: HQ828070], C group MKK gene *GhMKK5* [GenBank accession number: HQ637469] and D group MKK gene *GhMKK9* [GenBank accession number: HQ651069]) and *GhMPK4* were then inserted into the pGADT7 vector. The appropriate combinations of these recombinant plasmids were cotransformed into a yeast Y2H Gold strain and plated on DDO, QDO and QDO/X media.

To verify the interaction between GhMORG1 and GhMKK6, the ORFs of *GhMORG1* and *GhMKK6* were fused into pUC‐SPYCE‐35S and pUC‐SPYNE‐35S, respectively, and transformed into *A. tumefaciens* strain GV3101. The recombinant plasmids were cotransformed into *N. benthamiana* leaves using *Agrobacterium*‐mediated transient infection. The fluorescence from yellow fluorescent protein (YFP) was observed with an LSM 880 META confocal microscope (Carl Zeiss). To detect the interaction between GhMPK4 and GhMKK6 or GhMORG1, the ORF of *GhMPK4* was fused into pUC‐SPYCE‐35S and pUC‐SPYNE‐35S and cotransformed with GhMKK6 or GhMORG1, respectively.

### Agrobacterium‐mediated VIGS

*Agrobacterium*‐mediated VIGS was performed according to the methods of Gu *et al. *(2014). The fragment of *GhMORG1* or that of other genes in this study was inserted into the pCLCrV‐A vector. The primers used are shown in Table S9. The recombinant plasmids, pCLCrV‐A or pCLCrV‐B, were transformed into *A. tumefaciens* strain EHA105. The *A. tumefaciens* cells that contained pCLCrV‐A or pCLCrV‐B were mixed equally and inoculated into two fully expanded cotton cotyledons. The inoculated cotton plants were used for functional analysis after inoculation for three weeks. Each assay was performed with at least three independent biological replicates.

### Pathogen biomass assays

DNA from cotton or tobacco leaves was isolated using the DNAsecure Plant Kit (Tiangen, China). The amount of *F. oxysporum* DNA relative to the amount of cotton or tobacco DNA was determined via qPCR as described previously (Wang *et al.*, 2017). Disease index [log_2_ (CutA/SK11)] values were determined by subtracting the C_t_ values of the cutinase from those of SK11 (Wang *et al.*, 2017). At least three biological replicates were analysed for all of the samples.

### Phosphoproteomics and proteomics

Total protein from each sample was extracted using lysis buffer (8 m urea, 1% Triton‐100, 10 mm dithiothreitol and 1% protease inhibitor cocktail) with an ultrasonic processor. The protein was precipitated with cold 20% TCA for 2 h at −20 °C and redissolved in 8 m urea. The protein was digested overnight by trypsin (1:50 trypsin: protein mass ratio).

For phosphoproteomics, immobilized metal affinity chromatography (IMAC) techniques were used to enrich the phosphopeptides, and liquid chromatography–tandem mass spectrometry (LC‐MS/MS) were used to analyse the peptides or phosphopeptides at Jingjie PTM Biolab (Hangzhou, China). Peptide mixtures were first incubated with IMAC microsphere suspensions with vibration. The IMAC microspheres with enriched phosphopeptides were collected. To remove nonspecifically adsorbed peptides, the IMAC microspheres were washed sequentially with 50% acetonitrile/6% trifluoroacetic acid and 30% acetonitrile/0.1% trifluoroacetic acid. To elute the enriched phosphopeptides from the IMAC microspheres, elution buffer containing 10% NH_4_OH was added and the enriched phosphopeptides were eluted with vibration. The supernatant containing phosphopeptides was collected and lyophilized for LC‐MS/MS analysis.

With respect to the database queries, the resulting MS/MS data were processed using a MaxQuant search engine (v.1.5.2.8) at Jingjie PTM Biolab (Hangzhou, China). Tandem mass spectra were queried against the AD1_BGI database, which was downloaded from www.cottongen.org. The mass tolerance for precursor ions was set to 20 ppm in the first search and 5 ppm in the main search, and the mass tolerance for fragment ions was set to 0.02 Da. Queries were performed with full tryptic digestion and allowed a maximum of four missed cleavages. The false discovery rate (FDR) was adjusted to < 1%, and the minimum score for modified peptides was set to >40.

### Detection of phosphorylation levels via Phos‐tag SDS‐PAGE gel analysis

To confirm that the putative substrates were phosphorylated by GhMKK6, we isolated 8 of the 32 proteins in cotton and inserted them into the pPZP211‐Flag vector. The recombinant plasmids were cotransformed with activated GhMKK6 (GhMKK6EE) into cotton protoplasts. The activity of GhMKK6EE was detected previously (Wang *et al.*, 2017). To confirm that the putative substrates were phosphorylated by GhMPK4, we obtained activated GhMPK4 (GhMPK4GA) as described previously by replacing conserved Asp‐200 with Gly and Glu‐204 with Ala (Berriri *et al.*, 2012). The preparation and transformation of cotton protoplasts were performed as described by Wang *et al. *(2018). Total proteins were extracted using extraction buffer [50 mm Tris/HCl (pH 7.5), 150 mm NaCl, 1% (v/v) Triton X‐100, 0.1% (w/v) SDS, phosphatase and protease inhibitors]. The Phos‐tag SDS‐PAGE separating gel consisted of 8% acrylamide, 50 µm Phos‐tag acrylamide (ApexBio, Houston, TX), 357 mm Tris buffer (pH 8.8) and 100 µm MnCl_2_. The Phos‐tag SDS‐PAGE gel analysis was performed using Mn^2+^‐Phos‐tag SDS‐PAGE according to the manufacturer’s protocol.

## Accession numbers

Sequence data for the genes discussed in this paper can be found at Cotton Genome Project database (http://www.cottongen.org) under the following Unique Names: GhMORG1 (CotAD_21502 _BGI‐AD1_v1.0), GhMKK6 (CotAD_25213_BGI‐AD1_v1.0), GhMPK4 (CotAD_19088_BGI‐AD1_v1.0), GhMBD (CotAD_33461_BGI‐AD1_v1.0), GhbHLH122 (CotAD_59974_BGI‐AD1_v1.0), GhRPL5 (CotAD_29344_BGI‐AD1_v1.0), GheIF4A (CotAD_53993_BGI‐AD1_v1.0), GheIF4G (CotAD_56516_BGI‐AD1_v1.0), GhCML42 (CotAD_35634_BGI‐AD1_v1.0), GhKLCR1 (CotAD_19835_BGI‐AD1_v1.0), GhRIN4 (CotAD_42472_BGI‐AD1_v1.0) and Gh14‐3‐3 (CotAD_64613_BGI‐AD1_v1.0).

## Conflict of interest

The authors have no conflicts of interest to declare.

## Author contributions

C.W. and X.G. conceived the project and designed the experiments. C.W. and X.H. performed most of the experiments and acquired with the assistance of J.W., L.W. and D.G., C.W. and H.G. performed the bioinformatic analysis and wrote the article.

## Supporting information


**Figure S1** The detection of the epitope‐tagged fusions of GhMKK3‐AD (line 1), GhMKK5‐AD (line 2), GhMKK6‐AD (line 3) and GhMKK9‐AD (line 4) in yeast using western blots.
**Figure S2** Identification of *GhMORG1*‐silenced cotton or *GhMORG1*‐overexpressing tobacco plants.
**Figure S3** Silencing *GhMORG1* decreased the resistance of cotton to *F. oxysporum*.
**Figure S4** The interaction between GhMPK4 and GhMORG1 or GhMKK6 was confirmed by BiFC experiments.
**Figure S5** Silencing *GhMPK4* reduced the resistance of cotton to *F. oxysporum*.
**Figure S6** Pearson’s correlation coefficient between CRV::00 and CRV::GhMORG1 cotton plants infected or uninfected with *F. oxysporum*.
**Figure S7** Motif analysis of the identified phosphorylation site.
**Figure S8** GhMORG1 increased the phosphorylation level of candidate substrates in cotton protoplasts.
**Figure S9** The interaction between GhMPK4 and candidate substrates and the phosphorylation level of candidate substrates in cotton protoplasts expressing or not expressing GhMPK4GA.

**Table S1** List of identified proteins in cotton, as determined by proteomics.
**Table S2** List of identified phosphorylation sites and phosphoproteins, as determined by cotton phosphoproteomics.
**Table S3** Motif‐X analysis results of all of the identified phosphorylation sites.
**Table S4** List of increased‐ and decreased‐regulated phosphorylated proteins in CRV::00 cotton after *F. oxysporum* infection.
**Table S5** GO enrichment analysis for increased‐ and decreased‐regulated phosphorylated proteins in CRV::00 cotton after *F. oxysporum* infection.
**Table S6** List of increased‐ and decreased‐regulated phosphorylated proteins from CRV::GhMORG1 cotton after *F. oxysporum* infection vs CRV::00 cotton after *F. oxysporum* infection.
**Table S7** GO enrichment analysis for increased‐ and decreased‐regulated phosphorylated proteins from CRV::GhMORG1 cotton after *F. oxysporum* infection vs CRV::00 cotton after *F. oxysporum* infection.
**Table S8** List of putative substrates of the GhMORG1‐dependent GhMKK6‐GhMPK4 cascade.
**Table S9** Primers used in this study.
