# Supplementary material for: Scaffold protein GhMORG1 enhances the resistance of cotton to Fusarium oxysporum by facilitating the MKK6‐MPK4 cascade
Source: Plant Biotechnol J. 2019 Dec 20;18(6):1421–33. doi: 10.1111/pbi.13307 (PMC7206998; doi:10.1111/pbi.13307)
Supplement: Supplementary file 1 — Figure S1 The detection of the epitope‐tagged fusions of GhMKK3‐AD (line 1), GhMKK5‐AD (line 2), GhMKK6‐AD (line 3) and GhMKK9‐AD (line 4) in yeast using western blots. Figure S2 Identification of GhMORG1‐silenced cotton or GhMORG1‐overexpressing tobacco plants. Figure S3 Silencing GhMORG1 decreased the resistance of cotton to F. oxysporum. Figure S4 The interaction between GhMPK4 and GhMORG1 or GhMKK6 was confirmed by BiFC experiments. Figure S5 Silencing GhMPK4 reduced the resistance of cotton to F. oxysporum. Figure S6 Pearson’s correlation coefficient between CRV::00 and CRV::GhMORG1 cotton plants infected or uninfected with F. oxysporum. Figure S7 Motif analysis of the identified phosphorylation site. Figure S8 GhMORG1 increased the phosphorylation level of candidate substrates in cotton protoplasts. Figure S9 The interaction between GhMPK4 and candidate substrates and the phosphorylation level of candidate substrates in cotton protoplasts expressing or not expressing GhMPK4GA. [file PBI-18-1421-s002.pdf]

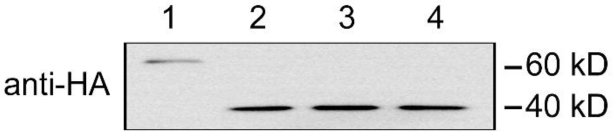

Figure S1. The detection of the epitope-tagged fusions of GhMKK3-AD (line 1), GhMKK5-AD (line 2), GhMKK6-AD (line 3) and GhMKK9-AD (line 4) in yeast using western blotting.

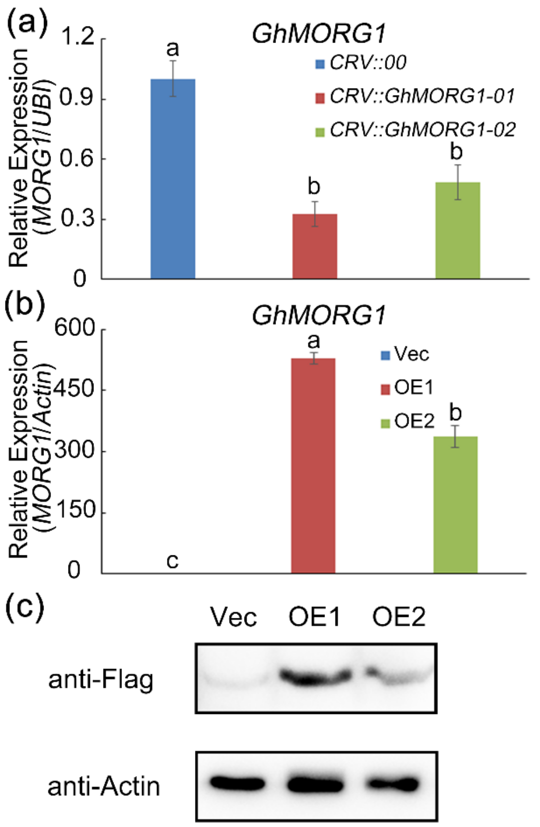

Figure S2. Identification of *GhMORG1*-silenced cotton or *GhMORG1*-overexpressing tobacco plants. **(a)**. Expression levels of *GhMORG1* in *GhMORG1*-silenced cotton. **(b and c)**. Expression levels or protein levels of GhMORG1 in *GhMORG1*-overexpression tobacco. Data are means ± SE of three independent experiments (n=15). Different letters indicate significant differences (P<0.01) based on Tukey's HSD test.

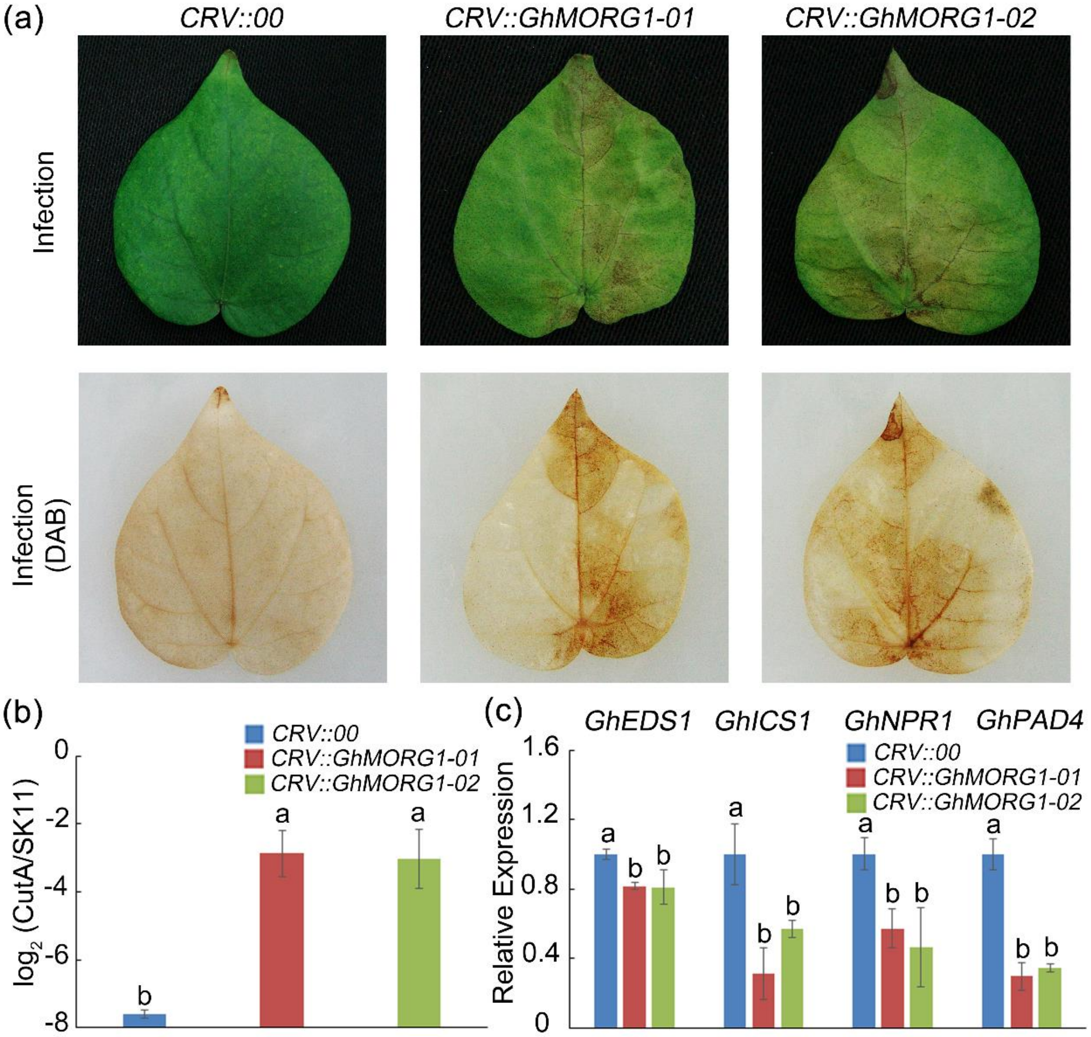

Figure S3. Silencing *GhMORG1* decreased the resistance of cotton to *F. oxysporum*. **(a).** Representative phenotypes of *GhMORG1*-silenced cotton plants after five days of *F. oxysporum* infection. **(b).** Pathogen disease index in *GhMORG1*-silenced cotton after five days of *F. oxysporum* infection. **(c).** Expression levels of SA-mediated defense pathway genes in CRV::00 and CRV::GhMORG1 cotton after five days of *F. oxysporum* infection. Data in (b and c) are means ± SE of three independent experiments (n = 15). Different letters indicate significant differences ($P < 0.01$) based on Tukey's HSD test.

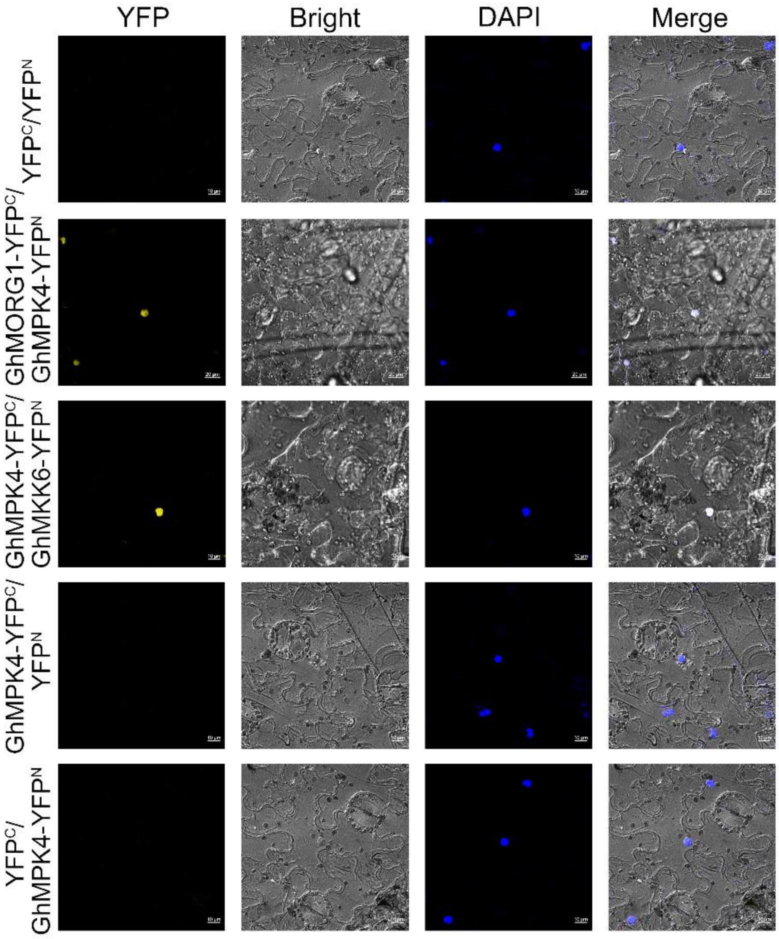

Figure S4. The interaction between GhMPK4 and GhMORG1 or GhMKK6 was confirmed by BiFC experiments.

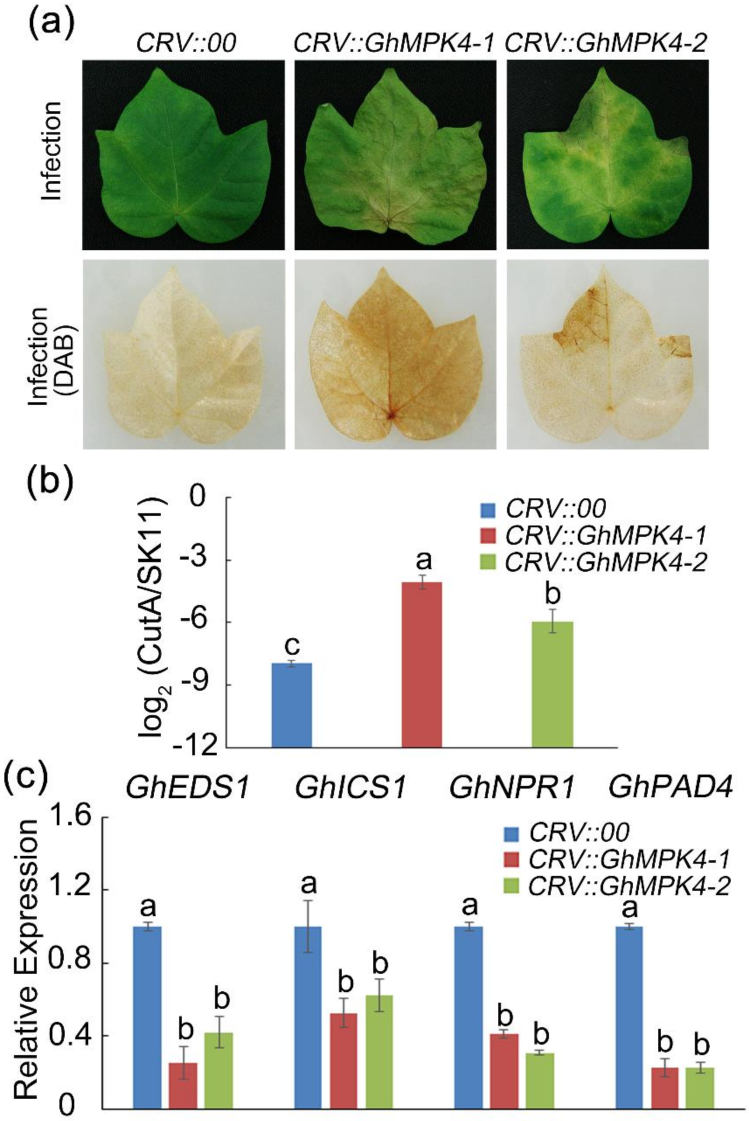

Figure S5. Silencing *GhMPK4* reduced the resistance of cotton to *F. oxysporum*. **(a).** Representative phenotypes of *GhMPK4*-silenced cotton after five days of *F. oxysporum* infection. **(b).** Pathogen disease index in *GhMPK4*-silenced cotton after five days of *F. oxysporum* infection. **(c).** Expression levels of SA-mediated defense pathway genes in CRV::00 and CRV::GhMPK4 cotton after five days of *F. oxysporum* infection. Data are means ± SE of three independent experiments (n = 15). Different letters indicate significant differences (P < 0.01) based on Tukey's HSD test.

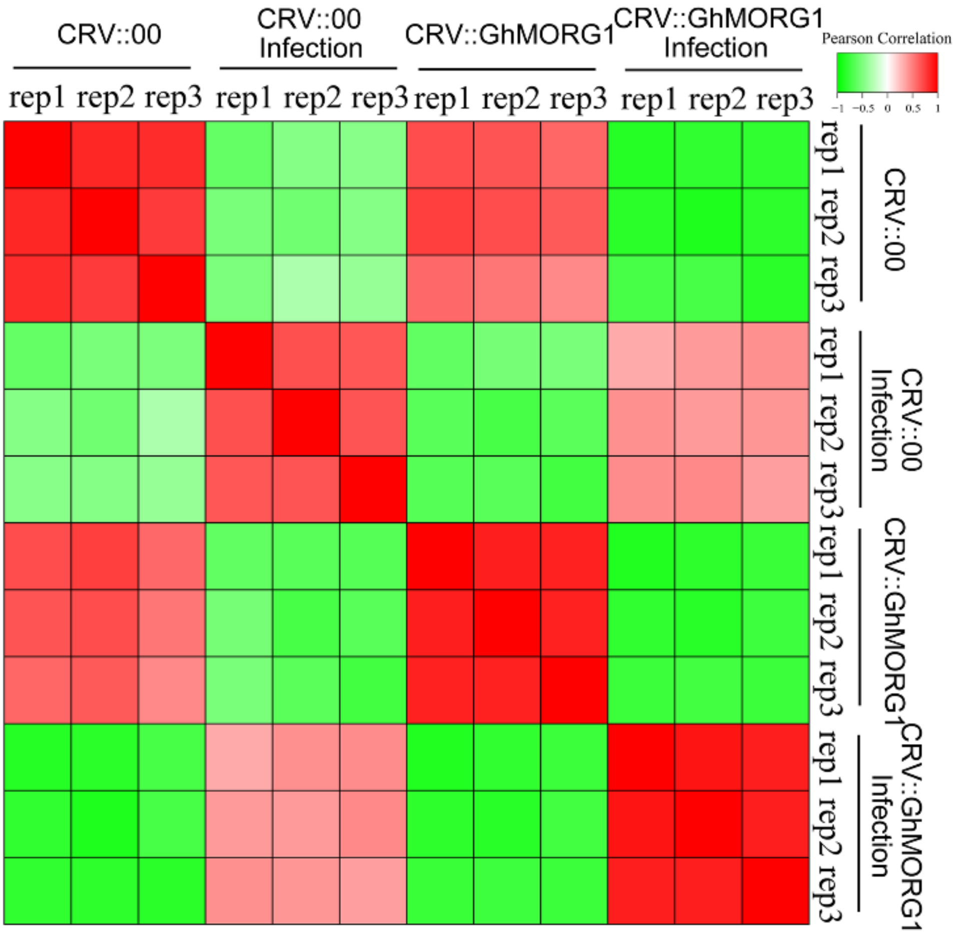

Figure S6. Pearson's correlation coefficient between CRV::00 and CRV::GhMORG1 cotton plants infected or uninfected with *F. oxysporum*.

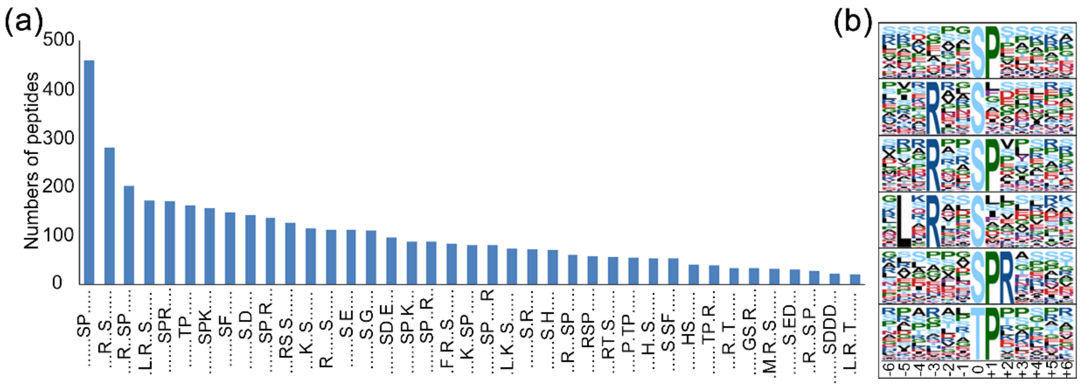

Figure S7. Motif analysis of the identified phosphorylation site. **(a)**. Number of identified phosphorylation sites in the indicated motif (. indicates any amino acid). **(b)**. The six most abundant phosphorylation motifs in this study. The height of each letter indicates the frequency of that amino acid in that position.

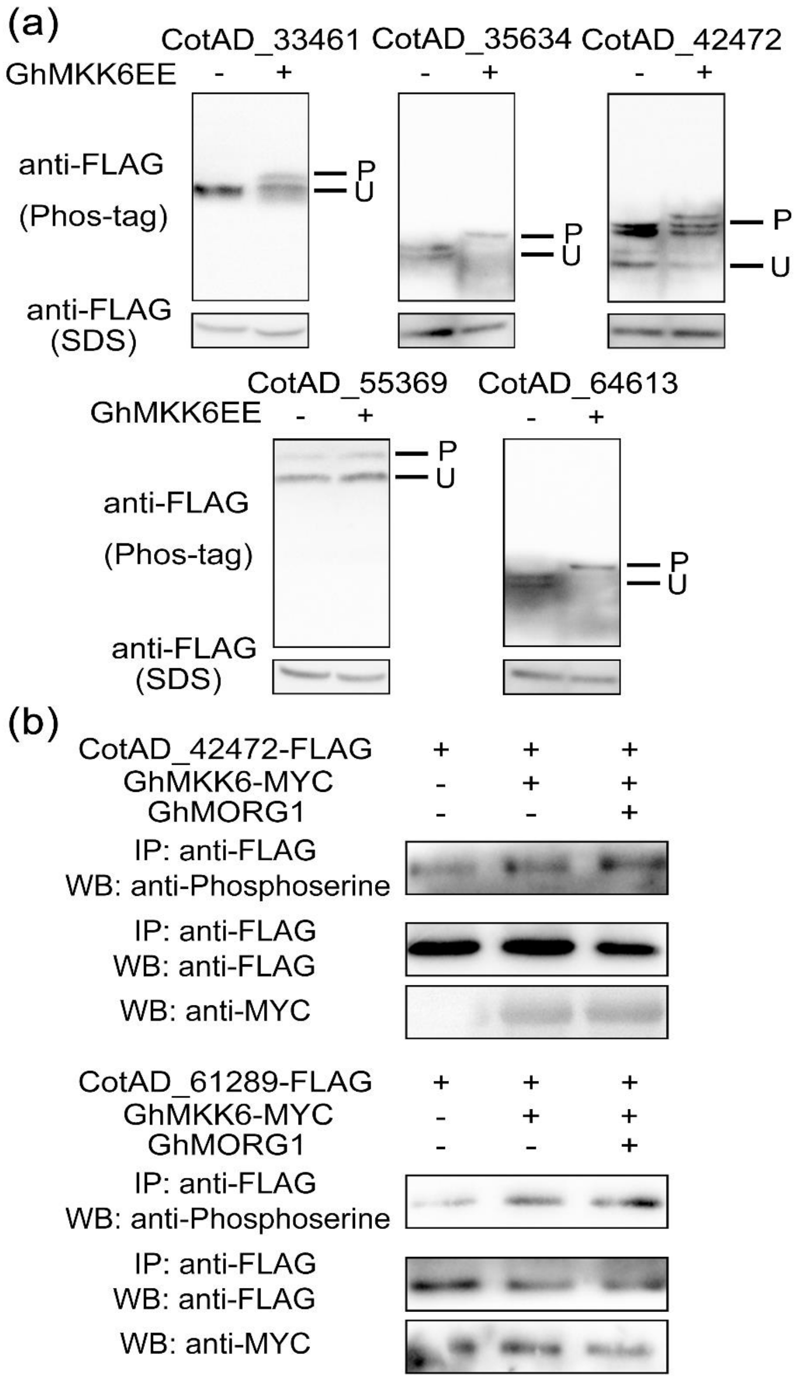

Figure S8. GhMORG1 increased the phosphorylation level of candidate substrates in cotton protoplasts. **(a)**. The phosphorylation level of candidate putative substrates in cotton protoplasts. At the top of each panel, the characters indicate the Gene ID in the Cotton Genome Project database. P and U indicate the phosphorylated and unphosphorylated forms, respectively. **(b)**. The phosphorylation level of candidate substrates in cotton protoplasts which co-expressed GhMKK6, GhMORG1 and substrates.

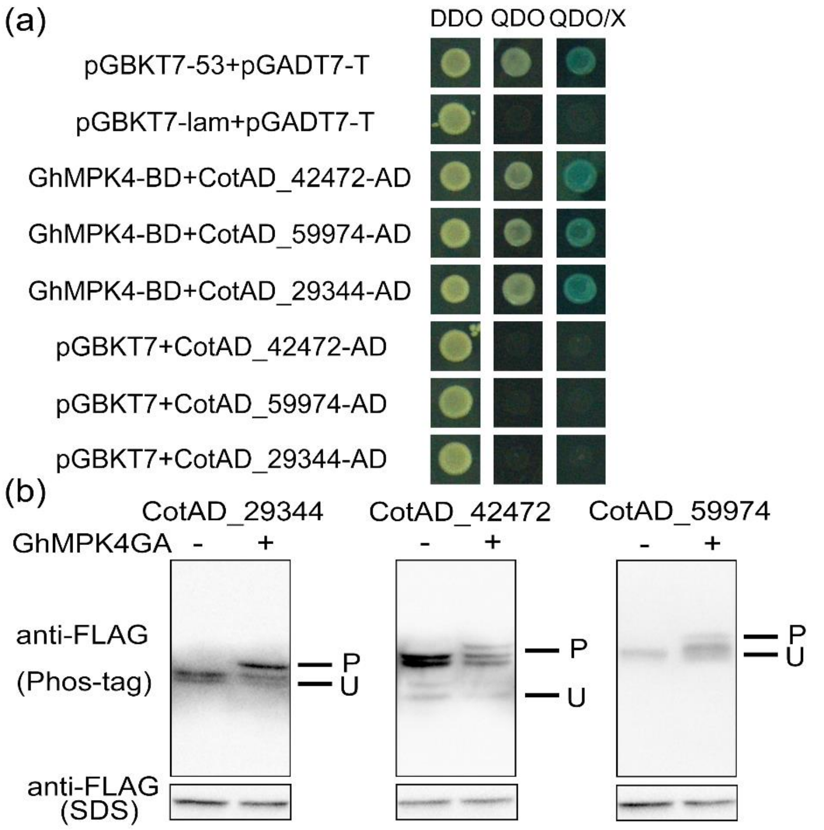

Figure S9. The interaction between GhMPK4 and candidate substrates and the phosphorylation level of candidate substrates in cotton protoplasts expressing or not expressing GhMPK4GA. **(a)**. The interaction between GhMPK4 and candidate substrates was confirmed by yeast two-hybrid. The indicated BD and AD fusion constructs were co-transformed into yeast and grown on DDO, QDO and QDO/X SD media. **(b)**. The phosphorylation level of candidate putative substrates in cotton protoplasts. At the top of each panel, the characters indicate the Gene ID in the Cotton Genome Project database. P and U indicate the phosphorylated and unphosphorylated forms, respectively.
